# SARS-CoV-2 Wave Two Surveillance in East Asia and the Pacific: Longitudinal Trend Analysis

**DOI:** 10.2196/25454

**Published:** 2021-02-01

**Authors:** Lori Ann Post, Jasmine S Lin, Charles B Moss, Robert Leo Murphy, Michael G Ison, Chad J Achenbach, Danielle Resnick, Lauren Nadya Singh, Janine White, Michael J Boctor, Sarah B Welch, James Francis Oehmke

**Affiliations:** 1 Buehler Center for Health Policy and Economics Feinberg School of Medicine Northwestern University Chicago, IL United States; 2 Feinburg School of Medicine Northwestern University Chicago, IL United States; 3 Institute of Food and Agricultural Sciences University of Florida Gainsville, FL United States; 4 Institute for Global Health Feinberg School of Medicine Northwestern University Chicago, IL United States; 5 Division of Infectious Disease Feinberg School of Medicine Northwestern University Chicago, IL United States; 6 International Food Policy Research Institute Washington DC, DC United States

**Keywords:** COVID-19, SARS-CoV-2, SARS-CoV-2 surveillance, second wave, wave two, wave 2, global COVID-19 surveillance, Asia Pacific public health surveillance, Asia Pacific COVID-19, Asian Pacific SARS-CoV-2, Asia Pacific surveillance metrics, dynamic panel data, generalized method of the moments, Asian Pacific econometrics, East Asian Pacific COVID-19 surveillance system, Pacific Asian COVID-19 transmission speed, Asian Pacific COVID-19 transmission acceleration, COVID-19 transmission deceleration, COVID-19 transmission jerk, COVID-19 7-day lag, Arellano-Bond estimator, generalized method of moments, GMM, Australia, Brunei, Cambodia, China, Fiji, French Polynesia, Guam, Indonesia, Japan, Kiribati, Laos, Malaysia, Mongolia, Myanmar, New Caledonia, Philippines

## Abstract

**Background:**

The COVID-19 pandemic has had a profound global impact on governments, health care systems, economies, and populations around the world. Within the East Asia and Pacific region, some countries have mitigated the spread of the novel coronavirus effectively and largely avoided severe negative consequences, while others still struggle with containment. As the second wave reaches East Asia and the Pacific, it becomes more evident that additional SARS-CoV-2 surveillance is needed to track recent shifts, rates of increase, and persistence associated with the pandemic.

**Objective:**

The goal of this study is to provide advanced surveillance metrics for COVID-19 transmission that account for speed, acceleration, jerk, persistence, and weekly shifts, to better understand country risk for explosive growth and those countries who are managing the pandemic successfully. Existing surveillance coupled with our dynamic metrics of transmission will inform health policy to control the COVID-19 pandemic until an effective vaccine is developed. We provide novel indicators to measure disease transmission.

**Methods:**

Using a longitudinal trend analysis study design, we extracted 330 days of COVID-19 data from public health registries. We used an empirical difference equation to measure the daily number of cases in East Asia and the Pacific as a function of the prior number of cases, the level of testing, and weekly shift variables based on a dynamic panel model that was estimated using the generalized method of moments approach by implementing the Arellano-Bond estimator in R.

**Results:**

The standard surveillance metrics for Indonesia, the Philippines, and Myanmar were concerning as they had the largest new caseloads at 4301, 2588, and 1387, respectively. When looking at the acceleration of new COVID-19 infections, we found that French Polynesia, Malaysia, and the Philippines had rates at 3.17, 0.22, and 0.06 per 100,000. These three countries also ranked highest in terms of jerk at 15.45, 0.10, and 0.04, respectively.

**Conclusions:**

Two of the most populous countries in East Asia and the Pacific, Indonesia and the Philippines, have alarming surveillance metrics. These two countries rank highest in new infections in the region. The highest rates of speed, acceleration, and positive upwards jerk belong to French Polynesia, Malaysia, and the Philippines, and may result in explosive growth. While all countries in East Asia and the Pacific need to be cautious about reopening their countries since outbreaks are likely to occur in the second wave of COVID-19, the country of greatest concern is the Philippines. Based on standard and enhanced surveillance, the Philippines has not gained control of the COVID-19 epidemic, which is particularly troubling because the country ranks 4th in population in the region. Without extreme and rigid social distancing, quarantines, hygiene, and masking to reverse trends, the Philippines will remain on the global top 5 list of worst COVID-19 outbreaks resulting in high morbidity and mortality. The second wave will only exacerbate existing conditions and increase COVID-19 transmissions.

## Introduction

### Background

**Figure 1:**
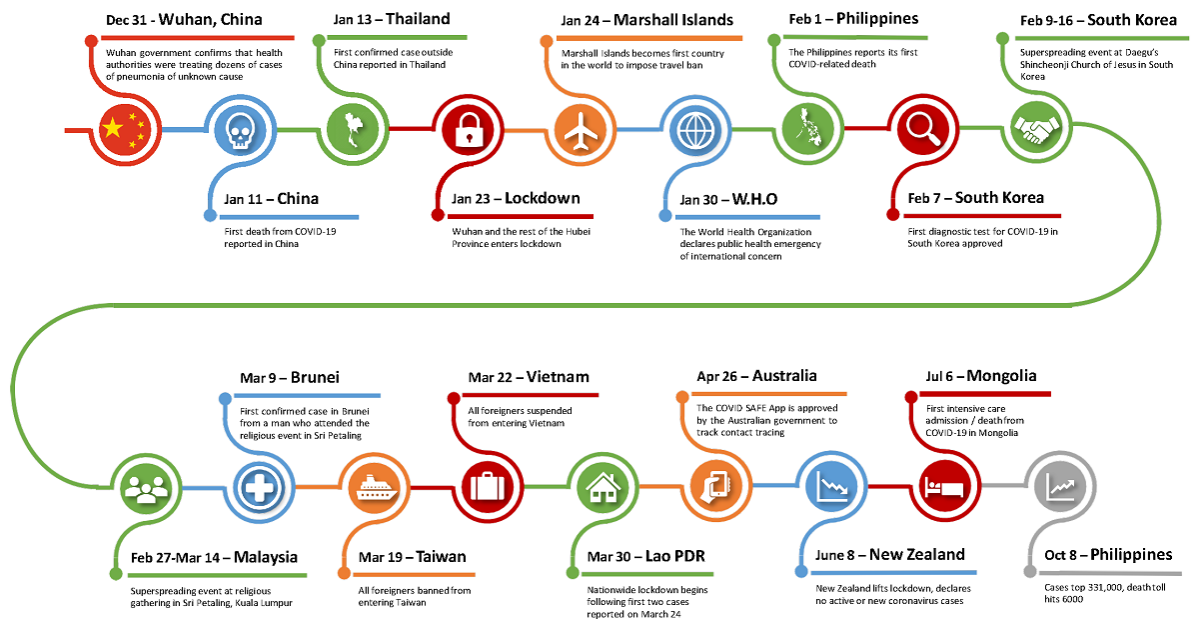
COVID-19 timeline in East Asia and the Pacific. WHO: World Health Organization. PDR: People's Democratic Republic.

COVID-19, caused by SARS-CoV-2, was first identified in Wuhan, Hubei Province, China, in December 2019 [1]. Since then, it has spread around the globe including to every country in the East Asia and Pacific region, severely straining governments, health care systems, economies, and quality of life globally (Figure 1). East Asia and the Pacific, as defined by the World Bank, consists of American Samoa, Australia, Brunei Darussalam, Cambodia, China, Fiji, French Polynesia, Guam, Hong Kong, Indonesia, Japan, Kiribati, People’s Democratic Republic of Korea, Republic of Korea, Lao People’s Democratic Republic, Macao, Malaysia, Marshall Islands, Federated States of Micronesia, Mongolia, Myanmar, Nauru, New Caledonia, New Zealand, Northern Mariana Islands, Palau, Papua New Guinea, the Philippines, Samoa, Singapore, Solomon Islands, Thailand, Timor-Leste, Tonga, Tuvalu, Vanuatu, and Vietnam [2]. Not all of these countries collect or report COVID-19 caseloads and deaths, such as North Korea. As of October 28, 2020, the World Health Organization (WHO) reports a total of 43,540,739 cases and 1,160,650 deaths in these countries [3]. This global region encompasses countries of diverse income levels, political systems, cultures, populations, geography, climate, and health care systems, factors which have profoundly influenced not only the effects of the virus but also the response of each member country.

### Government Response and Public Health Policy

The extreme quarantine implemented in Wuhan and the entire Hubei region of China mandated all residents to shelter in place without exception [4]. Wuhan imposed travel restrictions in and out of the city in addition to canceling gatherings, closing public places, and shutting down schools and universities [5]. China allocated significant resources for public health service and epidemic prevention and control [6].

The prompt response by East Asian countries was informed by the 2003 severe acute respiratory syndrome (SARS) and 2015 Middle East respiratory syndrome (MERS) outbreaks [7,8]. Singapore and Vietnam’s strategy of comprehensive surveillance to detect and contain as many cases as possible has been highly successful in controlling or eliminating SARS-CoV-2 [9,10]. Taiwan’s response to the 2003 SARS epidemic also informed COVID-19 response [11,12]. The Taiwan Centers for Disease Control aggressively traced confirmed cases while the government distributed masks and personal protection equipment [13,14]. Taiwan’s interconnected public health, medical, and insurance infrastructure reduces barriers to doctor appointments and follow-up visits, allowing their health care system to capture more cases. Furthermore, their single-payer model allows for centralized health records of population-level longitudinal data, a valuable tool for analyzing the spread of the pandemic [11].

South Korea was the hardest hit country outside of the Middle East during the MERS outbreak in 2015, prompting the Korea Centers for Disease Control (KCDC) to prepare for the next infectious disease outbreak [15]. When COVID-19 breached Korea’s borders, the KCDC actively performed contact tracing, quarantined exposed individuals, and diagnosed and isolated new cases with rapid and extensive testing [16-19].

New Zealand eradicated COVID-19 by introducing some of the strictest lockdown measures early on, allowing the government to pursue an elimination approach rather than the typical, mitigation-based model of pandemic planning. Schools and nonessential workplaces were closed, social gatherings banned, and severe travel restrictions applied [20-22]. Australia implemented similar though somewhat less stringent lockdown measures and border closures, resulting in substantially lower crude case fatality and hospitalization rates than many other high-income countries [23,24].

Some low- to middle-income countries such as Vietnam and Mongolia have also been able to implement successful responses to the pandemic despite limited resources or shared borders with China. As a result of Mongolia’s travel restrictions, lockdown measures, and surveillance of active infections, they reported no confirmed cases until March 10, 2020, and no intensive care admissions or deaths until July 6, 2020 [25]. Vietnam responded immediately to its first cases by activating an emergency prevention system involving intense surveillance, quarantine, and contact tracing. Those who broke social distancing measures were severely punished. As of July 8, 2020, the nation had the highest test per confirmed case ratio in the world [26-31]. This approach differs vastly from the widespread testing strategy employed by South Korea, which, although seen by many as a best practice in fighting the pandemic, is more resource intensive [32].

In contrast, the Philippines has become Southeast Asia’s coronavirus hotspot, overtaking Indonesia with a caseload exceeding 360,000 people with nearly 7000 deaths and no coherent strategy for defeating the virus [33]. Although their first case was reported in January 2020, a national lockdown was not enacted until March, and when citizens took to the streets to protest a lack of food and supplies 2 weeks later, President Rodrigo Duterte threatened that the police and military would shoot those who did not comply with stay-at-home orders [34-36]. Once the lockdown was lifted in June, cases quickly began to climb again. In addition, the Philippines was late to initiate COVID-19 measures including testing, isolation, and contact tracing [33]. Their result paralleled the United States with a similar outcome. The first wave is still raging through the islands while the second wave commences around the globe [37].

The Pacific Islands have been some of the least affected nations in the world due to their unique ability to shut down border traffic [38]. As of August 2, 2020, only 6 countries of the Pacific Islands (Papua New Guinea, Fiji, French Polynesia, Guam, New Caledonia, and Northern Mariana Islands) have recorded positive COVID-19 cases. Among the 12 countries without any confirmed cases, 10 are in this region (Kiribati, Marshall Islands, Micronesia, Nauru, Palau, Samoa, Solomon Islands, Tonga, Tuvalu, and Vanuatu). Furthermore, many nations recently strengthened their infectious disease prevention, surveillance, and response systems due to the re-emergence of measles in the area in 2019 [38-40].

### Health Systems, Vulnerable Populations, and Health Disparities

The East Asia and Pacific region encompass a wide range of health care systems with varying capacities for pandemic preparedness. In New Zealand, approximately one-fifth of the government’s spending goes to the health sector, and health services are either free or heavily subsidized [41]. In contrast, some of the world’s smallest, least developed, and most isolated nations in need of health system strengthening are in the Pacific Islands region [42].

In the Philippines, 43% of the urban population lives in slums, and people living in densely populated urban slums are unlikely to have the space or economic means to practice social distancing [43,44]. The majority of Filipinos pay out of pocket for health care, a prohibitive cost that disproportionately affects the country’s 7.5 million senior citizens, many of whom live in rural areas [45]. These barriers to access are exacerbated by the fact that hospitals in the Philippines are currently overwhelmed and reaching maximum capacity [45].

In Cambodia, more than 3 million people lack access to safe water, and 6 million lack access to improved sanitation. This disproportionately affects rural communities, where approximately 77% of Cambodians live, placing them at greater risk from the pandemic [46,47].

Meanwhile, the pandemic has added an additional layer of complexity to Indonesia’s longstanding problem of food insecurity and reliance on food imports. In 2018, 55% of the Indonesian population experienced moderate or severe food insecurity. Unemployment and other loss of income associated with the pandemic have likely exacerbated this problem, with 70% of low-income households reporting a loss of income and about the same proportion reporting shortages of some foods or not eating as much as they should, placing 24 million children at risk of food insecurity [48].

Myanmar has attempted to combat the aggravating effects of food insecurity on its already underfunded health sector by providing emergency rations through strategies such as community-based food banks [49,50], although 10%-15% of the population report consuming reduced quantities of nutritious foods [51].

Individuals with underlying medical conditions are also known to be at greater risk of COVID-19. In Australia, Indigenous Australians constitute a uniquely vulnerable population due to the increased prevalence of diabetes and respiratory and cardiovascular conditions, as well as high reported smoking rates [52,53]. China is notorious for having the worst air pollution problem in the world, which may correlate with susceptibility to respiratory infections [54-56].

Despite early exposure, a high population density, an aging population, and little social distancing measures, Japan reports low rates of infection and death from COVID-19 [57,58]. This has led to some hypotheses that the Bacille Calmette-Guérin (BCG) vaccine against tuberculosis may protect against the virus, as countries that mandate the BCG vaccine have relatively low per capita death rates from COVID-19 [57,59].

### Economy

Tourism is an important source of revenue for many economies in developing Asia and the Pacific [50,60,61]. For countries like Palau, where international tourism receipts are close to 50% of the GDP (gross domestic product) and over a third of international tourists are from China, the decline in tourism due to COVID-19 has been devastating [60].

China is a major trade destination for many developing Asian economies such as Mongolia, the Philippines, Singapore, Taipei, and Vietnam [60]. Long quarantine-like conditions have the potential to deeply harm export-based economies such as Mongolia, where coal exports were reduced due to border restrictions [25].

### Culture

While Western cultures endorse individualism and a more independent self-concept, Eastern cultures emphasize collectivism and a concept of the self as interdependent with others, which may motivate individuals to remain committed to COVID-19 precautions even at the expense of personal freedoms [62]. Some of the actions that have enabled Asian countries to contain the spread of the virus have been challenging to Western notions of privacy and individual freedom; such measures have been almost universally accepted in Asian countries [26,32]. Therefore, interpersonal transmission of the virus may be less likely in East Asian countries [63].

Public health departments, as well as universities and media outlets, are tracking the novel coronavirus using raw data, including the number of new infections, testing, positivity, R_0_ (reproduction number), deaths, local hospital capacity, etc [39,64-93]. Public health surveillance informs policy on “flattening the curve” of COVID-19 [94-97]. Epidemiologists have utilized various modeling techniques to forecast the numbers of cases and deaths attributed to the virus [98-102]. Both the WHO and the Center for Systems Science and Engineering at Johns Hopkins University have developed tracking tools [98]. While helpful, these static metrics suffer from incomplete case ascertainment and data contamination [94,96]. Existing surveillance is a proxy for the true coronavirus caseload because public health surveillance systems tend to pick up the most severe cases [103,104], which is especially problematic when tracking SARS-CoV-2 because most carriers are asymptomatic, presymptomatic, or only have mild symptoms [105-108]. Public health surveillance that can control for these limitations are needed. Moreover, metrics that detect how transmission speed of the novel coronavirus, shifts in the pandemic, acceleration in speed, and persistence of COVID-19 based on prior infections are needed to supplement existing measures [83].

### Objective

The objective of our research is to use a longitudinal trend analysis study design in concert with dynamic panel modeling and method of moments to correct for existing surveillance data limitations [94,96]. Specifically, we will measure significant weekly shifts in the increase, decrease, or plateaued transmission of SARS-CoV-2. Our study will measure the underlying causal effect from last week that persists through this week, with a 7-day persistence rate to explain a clustering/declustering effect. The 7-day persistence rate represents an underlying disease transmission wave, where a large number of transmissions that resulted in a large number of infections today then “echoes” forward into a large number of new transmissions and hence a large number of new cases 7 days later. If positive, it is consistent with, for example, a mega-event (eg, the large religious gathering at Daegu’s Shincheonji Church of Jesus in South Korea) that causes an increase in the number of cases in adjoining days, among other explanations [16]. If zero or nonsignificant, it is indicative of a constant rate of new infections and/or a constant size of the infectious population. In summary, we will measure negative and positive shifts in the transmission of SARS-CoV-2 or acceleration/deceleration rates that are not limited by sampling bias.

## Methods

We conducted a longitudinal trend analysis for our study design using data extracted from the internet. The COVID Tracking Project [109], Our World in Data [110], and The Foundation for Innovative New Diagnostics [111] compiles data from multiple sources on the web [112]; data for the most recent 4 weeks were accessed from the GitHub repository [113-115]. This resulted in a panel of 26 countries in East Asia and the Pacific with 30 days in each panel (n=780). An empirical difference equation was specified in which the number of positive cases in each country for each day is a function of the prior number of cases, the level of testing, and weekly shift variables that measure whether the contagion was growing faster, at the same rate, or slower than the previous weeks. This resulted in a dynamic panel model that was estimated using the generalized method of moments (GMM) approach by implementing the Arellano-Bond estimator in R (The R Foundation) [94,96,116]. Additionally, we report on the novel dynamic surveillance metrics of speed, acceleration, and jerk [94,96].

## Results

### Country Regression Results

**Figure 2:**
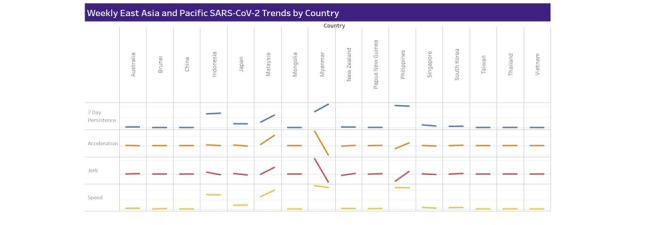
COVID-19 weekly trends in East Asia and the Pacific [117].

Regression results are presented for 26 East Asian and Pacific countries in Table 1. Weekly surveillance data in Tables 2-6 and Figure 2 [117] are based on these regressions.

The Wald statistic for the regression was significant (*χ*^2^_5_=49,836,424; *P*<.001). The Sargan test was not significant, failing to reject the validity of overidentifying restrictions (*χ*^2^_294_=18; *P*=.99).

**Table 1:** Arellano-Bond dynamic panel data modeling of the number of daily infections reported by country in East Asia and the Pacific, October 5-18, 2020.

Variable	Statistic	*P* value
L1Pos^a^	*r*=–0.007	.89
L7Pos^b^	*r*=0.887	<.001
Cumulative tests	*r*=0.000	.15
Weekend	*r*=–0.603	<.001
Wald statistic for regression	*χ*^2^_5_=49836424	<.001
Sargan statistic for validity	*χ*^2^_294_=18	.99

^a^L1Pos: the statistical impact of a 1-day lag of speed on today’s value of speed.

^b^L7Pos: the statistical impact of the 7-day lag of speed on today’s value of speed. New cases per day tend to have an echo effect 7 days later. Reported as the weekly average number of new cases per day that are attributable to the weekly average of the 7-day lag of the number of new cases per day.

Table 2, for the week of October 5-11, and Table 3, for the week of October 12-18, present traditional surveillance metrics including new COVID-19 cases, cumulative COVID-19 cases, 7-day moving average of COVID-19 infections, infections per 100,000 population, deaths, cumulative deaths, and 7-day moving average of deaths rates per 100,000 population. Overall, in East Asia and the Pacific, by the second week, there were 9951 new daily cases of COVID-19, 1,076,042 cumulative cases of COVID-19, a 7-day moving average of 8005, an infection rate per 100,000 population of 0.4344, 216 daily deaths, and 28,053 cumulative deaths.

For the week of October 5-11, Indonesia led the East Asian and Pacific region with the highest number of new cases at 4294, followed by the Philippines at 2156 and Myanmar at 2158. The number of deaths follow the same pattern for Indonesia, the Philippines, and Myanmar at 88, 86, and 32 deaths, respectively.

For the week of October 12-18 (Table 3), Indonesia, the Philippines, and Myanmar continued to lead the region with 4301, 2588, and 1287 new COVID-19 infections and 84, 72, and 39 deaths, respectively. The cumulative number of deaths for the region reached 28,053 by the second week in October.

**Table 2:** Static surveillance metrics for the week of October 5-11, 2020.

Country	New COVID-19 cases, n	Cumulative COVID-19 cases, n	7-day moving average of new cases	Rate of infection	New deaths, n	Cumulative deaths, n	Death rate per 100k
Australia	15	27,244	17.57	0.06	0	897	0
Brunei	0	146	0	0	0	3	0
China	21	90,778	24.86	0	0	4739	0
French Polynesia	0	2692	104	0	0	10	0
Guam	89	3078	54.14	53.20	2	60	1.20
Indonesia	4294	328,952	4207	1.59	88	11,765	0.03
Japan	679	88,912	510.43	0.54	3	1627	0
Malaysia	374	15,096	429.71	1.17	3	155	0.01
Mongolia	0	318	0	0	3	0	0.09
Myanmar	2158	26,064	1365.86	3.99	32	598	0.06
New Zealand	1	1515	2.43	0.02	0	25	0
Papua New Guinea	0	549	1.29	0	0	7	0
Philippines	2156	336,926	2514	1.99	86	6238	0.08
Singapore	7	1,010,010	9.43	0.12	0	27	0
South Korea	58	57,866	73.57	0.11	2	432	0
Taiwan	0	24,606	1.43	0	0	7	0
Thailand	0	527	7.29	0	0	59	0
Vietnam	2	3634	1.57	0	0	35	0
Region	9854	1107	7999	0.43	219	26,684	0.01

**Table 3:** Static surveillance metrics for the week of October 12-18, 2020.

Country	New COVID-19 cases, n	Cumulative COVID-19 cases, n	7-day moving average of new cases	Rate of infection	New deaths, n	Cumulative deaths, n	Death rate per 100k
Australia	12	27,383	19.86	0.05	0	904	0
Brunei	0	147	0.14	0	0	3	0
China	30	90,955	25.29	0	0	4739	0
French Polynesia	62	3797	157.9	22.20	0	14	0
Guam	0	3617	77.00	0	3	66	1.79
Indonesia	4301	357,762	4115.71	1.59	84	12,431	0.03
Japan	593	92,656	534.9	0.47	9	1670	0.01
Malaysia	869	19,627	647.3	2.72	4	180	0.01
Mongolia	0	324	0	0	4	0	0.12
Myanmar	1387	34,875	1258.71	2.57	39	838	0.07
New Zealand	3	1530	2.14	0.06	0	25	0
Papua New Guinea	3	581	4.57	0.03	0	7	0
Philippines	2588	354,338	2487.4	2.39	72	6603	0.07
Singapore	3	57,904	5.43	0.05	0	28	0
South Korea	91	25,199	84.71	0.18	1	444	0
Taiwan	0	535	1.14	0	0	7	0
Thailand	7	3686	7.43	0.01	0	59	0
Vietnam	2	1126	2.71	0	0	35	0
Region	9951	1,076,042	8005	0.44	216	28,053	0.01

Tables 4 and 5 provide the novel surveillance metrics for the weeks of October 5-11 and October 12-18, respectively. During the week of October 5-11 (Table 4), French Polynesia had the highest speed or velocity of new cases at 37 per 100,000 population, followed by Guam at 32.6 cases per 100,000 population. The highest rates of acceleration per 100,000 population was 0.312 for Myanmar, 0.025 for Malaysia, and 0.015 for Indonesia. The highest jerk rates were 0.228, 0.027, and 0.008 for Myanmar, Indonesia, and Japan, respectively. French Polynesia and Guam ranked 1 and 2 for 7-day persistence at 28.68 and 25.14, respectively, meaning these cases were statically attributed to those persons infected 7 days earlier.

**Table 4:** Novel surveillance metrics for the week of October 5-11, 2020.

Country	Speed^a^	Acceleration^b^	Jerk^c^	7-day persistence effect on speed^d^
Australia	0.07	0	0	0.05
Brunei	0	0	0	0
China	0	0	0	0
French Polynesia	37.24	0	–11.66	28.69
Guam	32.36	–5.12	–9.82	26.14
Indonesia	1.55	0.02	0.03	1.32
Japan	0.40	0.01	0.01	0.37
Malaysia	1.34	0.03	0	0.52
Mongolia	0	0	0	0
Myanmar	2.53	0.31	0.23	1.53
New Zealand	0.05	–0.01	–0.02	0.05
Papua New Guinea	0.01	0	0	0.01
Philippines	2.33	–0.07	–0.11	2.12
Singapore	0.17	0	0	0.26
South Korea	0.14	0	0	0.12
Taiwan	0.01	0	0	0
Thailand	0.01	0	0	0.01
Vietnam	0	0	0	0
Region	0.41	0.01	0	0.33

^a^Daily positives per 100k (weekly average of new daily cases per 100k).

^b^Day-to-day change in the number of positives per day, weekly average, per 100k.

^c^Week-over-week change in acceleration, per 100k.

^d^New cases per day per 100k attributed to new cases 7 days ago.

Table 5 presents the novel surveillance metrics for the second week of our study period. Between October 12 and 18, French Polynesia ranked first in speed of new infections at 56.5 per 100,000, followed by Guam at 46 per 100,000. French Polynesia and Guam are several standard deviations higher than the rest of the East Asian and Pacific region. French Polynesia had the highest acceleration rate at 3.17 per 100,000 population, and Polynesia had the highest positive jerk at 15.4 per 100,000 population. French Polynesia and Guam had the largest 7-day persistence during the October 12-18 period (Table 6). In summary, French Polynesia, Malaysia, the Philippines, South Korea, and New Zealand have positive speeds, acceleration, jerks, and 7-day persistence, indicating an upwards shift in the pandemic.

**Table 5:** Novel surveillance metrics for the week of October 12-18, 2020.

Country	Speed^a^	Acceleration^b^	Jerk^c^	7-day persistence effect on speed^d^
Australia	0.08	0	0.01	0.06
Brunei	0.03	0	0	0
China	0	0	0	0
French Polynesia	56.52	3.17	15.45	33.04
Guam	46.03	–7.60	–19.13	28.72
Indonesia	1.52	0	–0.01	1.38
Japan	0.42	–0.01	–0.01	0.36
Malaysia	2.03	0.22	0.10	1.19
Mongolia	0	0	0	0
Myanmar	2.33	–0.20	–0.12	2.24
New Zealand	0.04	0.01	0.01	0.04
Papua New Guinea	0.05	0	0	0.01
Philippines	2.30	0.06	0.04	2.06
Singapore	0.10	–0.01	–0.01	0.15
South Korea	0.16	0.01	0.01	0.13
Taiwan	0	0	0	0.01
Thailand	0.01	0	0	0.01
Vietnam	0	0	0	0
Region	0.41	0	0	0.36

^a^Daily positives per 100k (weekly average of new daily cases per 100k).

^b^Day-to-day change in the number of positives per day, weekly average, per 100k.

^c^Week-over-week change in acceleration, per 100k.

^d^New cases per day per 100k attributed to new cases 7 days ago.

**Table 6:** Seven-day persistence difference.

Country	7-day persistence	
	October 11, 2020	October 18, 2020
French Polynesia	28.69	33.04
Guam	26.14	28.72
Philippines	2.12	2.06
Myanmar	1.53	2.24
Indonesia	1.32	1.38

The most populous countries in East Asia and Pacific include China, Indonesia, Japan, Philippines, and Vietnam (Table 7). Countries with larger populations are at risk for having more COVID-19 infections by virtue of size, but this was not necessarily the case when comparing population size to the speed, acceleration, jerks, and 7-day persistence in Tables 4 and 5.

For comprehensive surveillance of static or traditional surveillance metrics with novel surveillance metrics for East Asia and Pacific, see Multimedia Appendices 1-3.

**Table 7:** Most populous East Asian countries.

Country^a^	Population as of 2020, N
China	1,439,323,776
Indonesia	273,523,615
Japan	126,476,461
Philippines	109,581,078
Vietnam	97,338,579

^a^Does not include countries that do not track or report COVID-19 cases (eg, North Korea).

## Discussion

Countries in the East Asia and Pacific region have had differential success in combating the COVID-19 pandemic, with some countries among the most successful in the world at containing the pandemic and others in serious jeopardy. China, Taiwan, South Korea, and Japan had early outbreaks that were successfully contained through stringent protective measures. Some of the smaller islands in the Pacific encountered the disease much later than other countries, but after initial exposure, French Polynesia and Malaysia had outbreaks that swiftly affected their nations.

While South Korea, New Zealand, and Australia are demonstrating some increases in speed, acceleration, jerk, and 7-day persistence, these nations had successfully implemented COVID-19 control policies that literally eliminated COVID-19. However, as wave two of the COVID-19 pandemic has recently began the cycle of transmissions, these countries had either low caseloads or zero caseloads; hence, any new cases are going to result in increased rates of transmission. The new cases in countries that previously eradicated COVID-19 indicate a need to reinstate public health guidelines to keep the second wave of COVID-19 transmission from gaining a larger foothold. Moreover, other countries in East Asia and the Pacific who are presently dealing with the first wave will have novel infections from wave two as well, such as Indonesia and the Philippines who have the highest cumulative infections and cumulative deaths from COVID-19. While the Philippines was decelerating during the week of October 5-11, it reversed course and now has a positive acceleration.

The region’s biggest successes are Singapore, New Zealand, Vietnam, South Korea, and China. In theory, it is likely easier to control for outbreaks in island nations such as Singapore and New Zealand; however, not all islands experienced their level of prevention and mitigation. Moreover, relative to population size (Table 7), South Korea, Vietnam, and China experienced an initial COVID-19 outbreak and took preventative measures to control further spread successfully.

## Appendix

Multimedia Appendix 1 Weekly East Asia and Pacific 7-day persistence map.

Multimedia Appendix 2 Weekly East Asia and Pacific acceleration jerk map.

Multimedia Appendix 3 Weekly East Asia and Pacific statistics.

## Abbreviations

BCG: Bacille Calmette-Guérin
GDP: gross domestic product
GMM: generalized method of moments
KCDC: Korean Centers for Disease Control
MERS: Middle East respiratory syndrome
SARS: severe acute respiratory syndrome
WHO: World Health Organization
