# Supplementary figures and images for: SARS-CoV-2 Wave Two Surveillance in East Asia and the Pacific: Longitudinal Trend Analysis
Source: J Med Internet Res. 2021 Feb 1;23(2):e25454. doi: 10.2196/25454 (PMC7857528; doi:10.2196/25454)

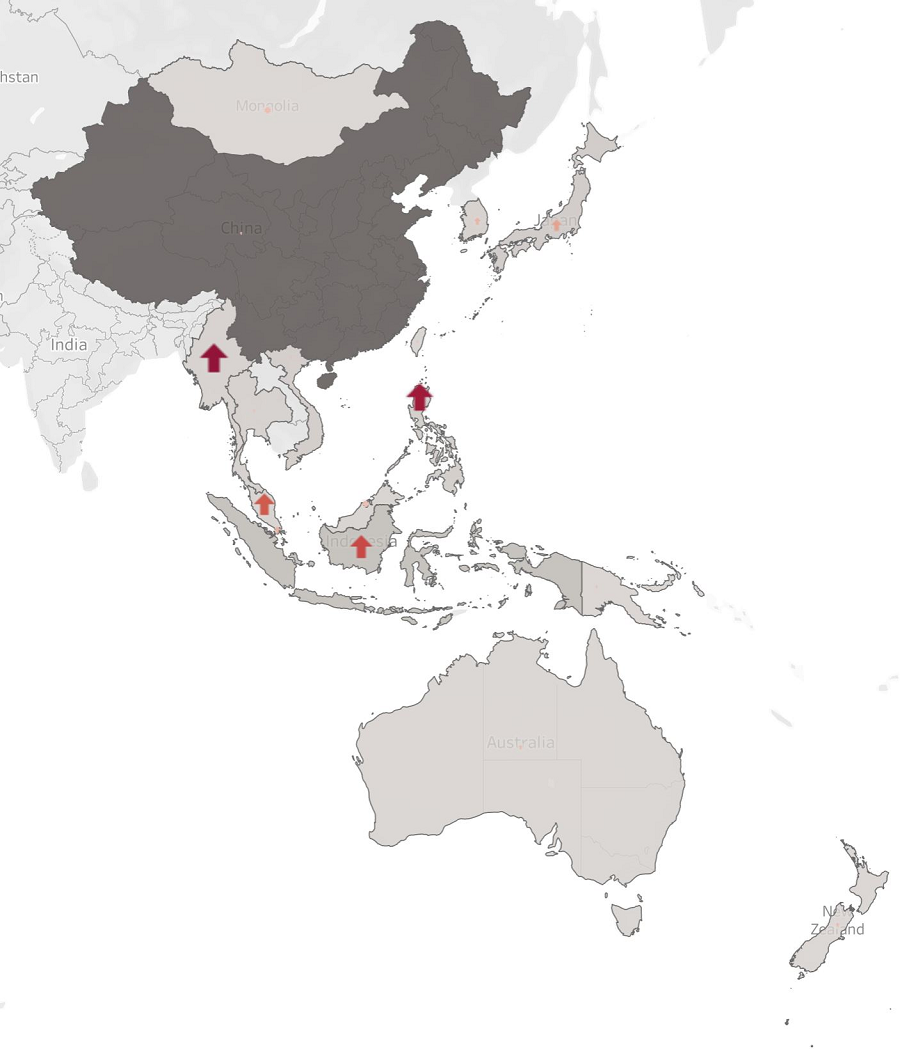

Supplement: Multimedia Appendix 1 [file jmir_v23i2e25454_app1.png]

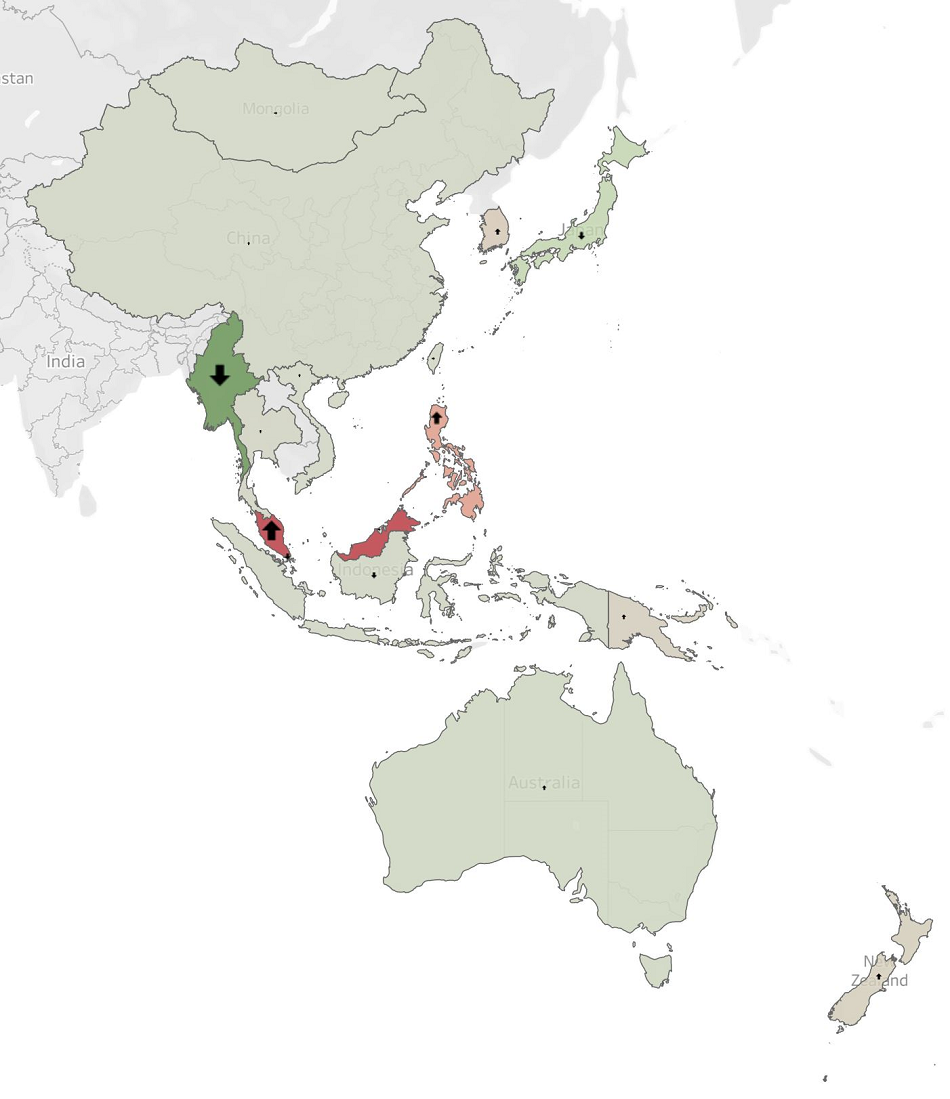

Supplement: Multimedia Appendix 2 [file jmir_v23i2e25454_app2.png]

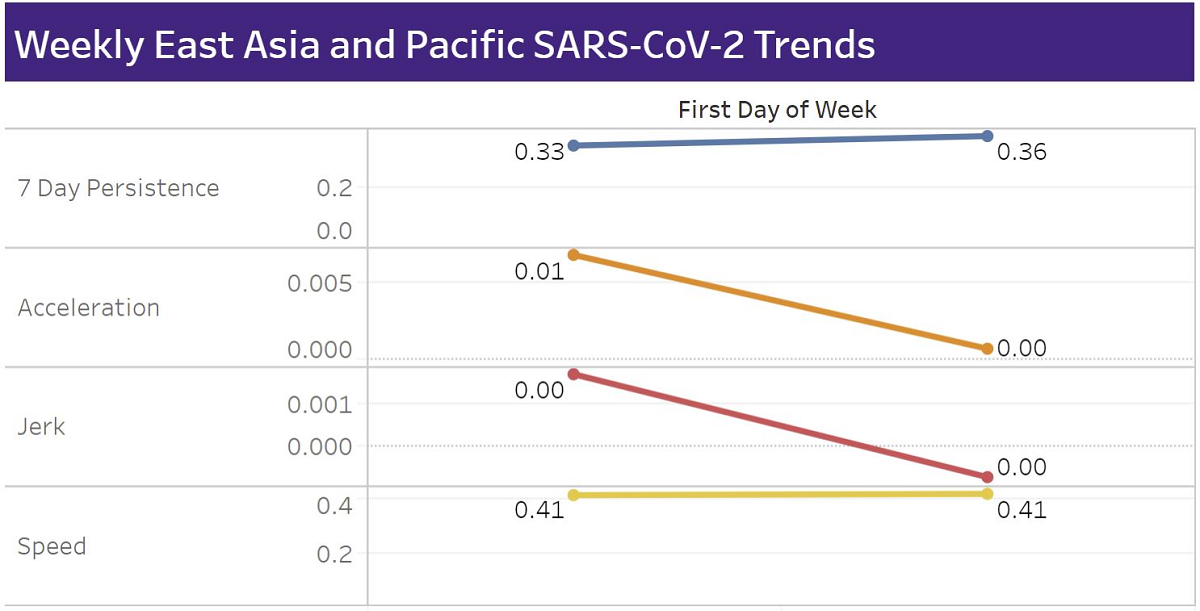

Supplement: Multimedia Appendix 3 [file jmir_v23i2e25454_app3.png]
